# Supplementary material for: Modulation of Human Hsp90α Conformational Dynamics by Allosteric Ligand Interaction at the C-Terminal Domain
Source: Sci Rep. 2019 Feb 7;9:1600. doi: 10.1038/s41598-018-35835-0 (PMC6367426; doi:10.1038/s41598-018-35835-0)
Supplement: Supplementary file 1 — Supporting Information [file 41598_2018_35835_MOESM1_ESM.pdf]

Modulation of Human Hsp90α Conformational Dynamics by Allosteric Ligand Interaction at the C-Terminal Domain

David L. Penkler[1] and Özlem Tastan Bishop[1]*

[1]Research Unit in Bioinformatics (RUBi), Department of Biochemistry and Microbiology, Rhodes University, Grahamstown, 6140, South Africa

[*] Correspondence should be addressed to (email: o.tastanbishop@ru.ac.za)

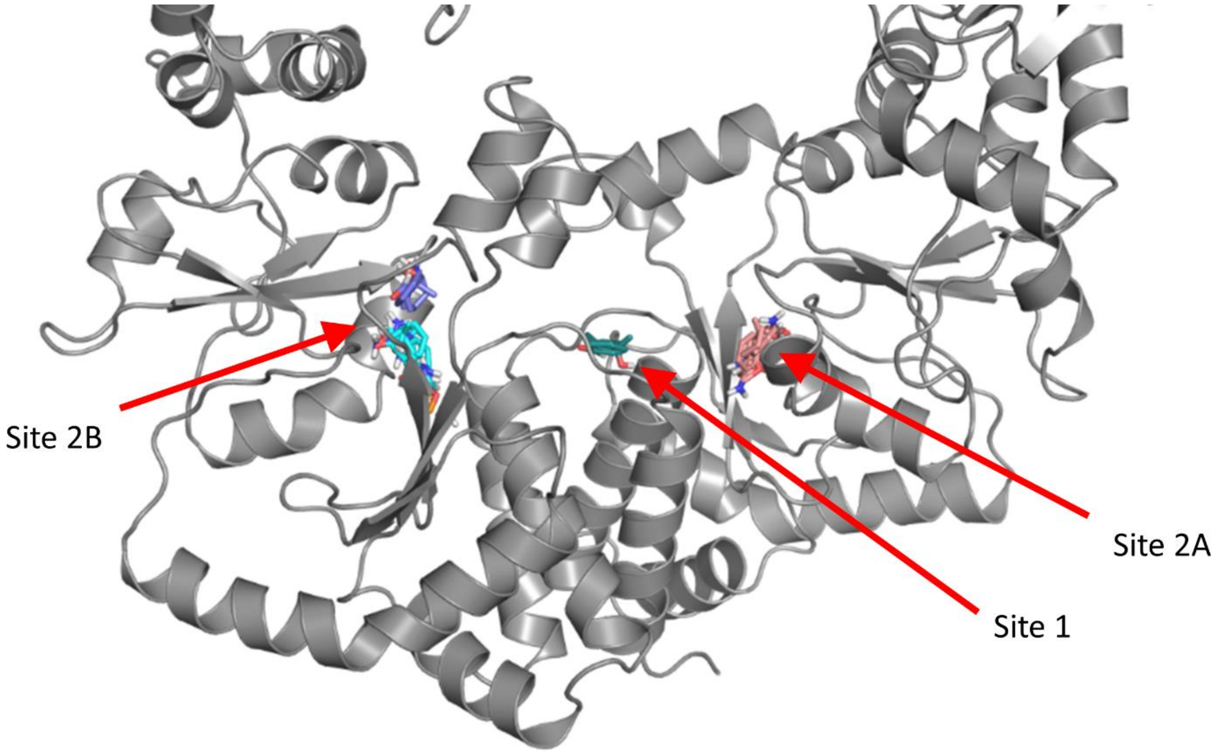

Figure S1: FTMap unbiased screen reveals two putative ligand binding sites in the CTD. Site 1 is located at the four-helix bundle. Site 2 is mirrored in both protomers site 2A and 2B respectively.

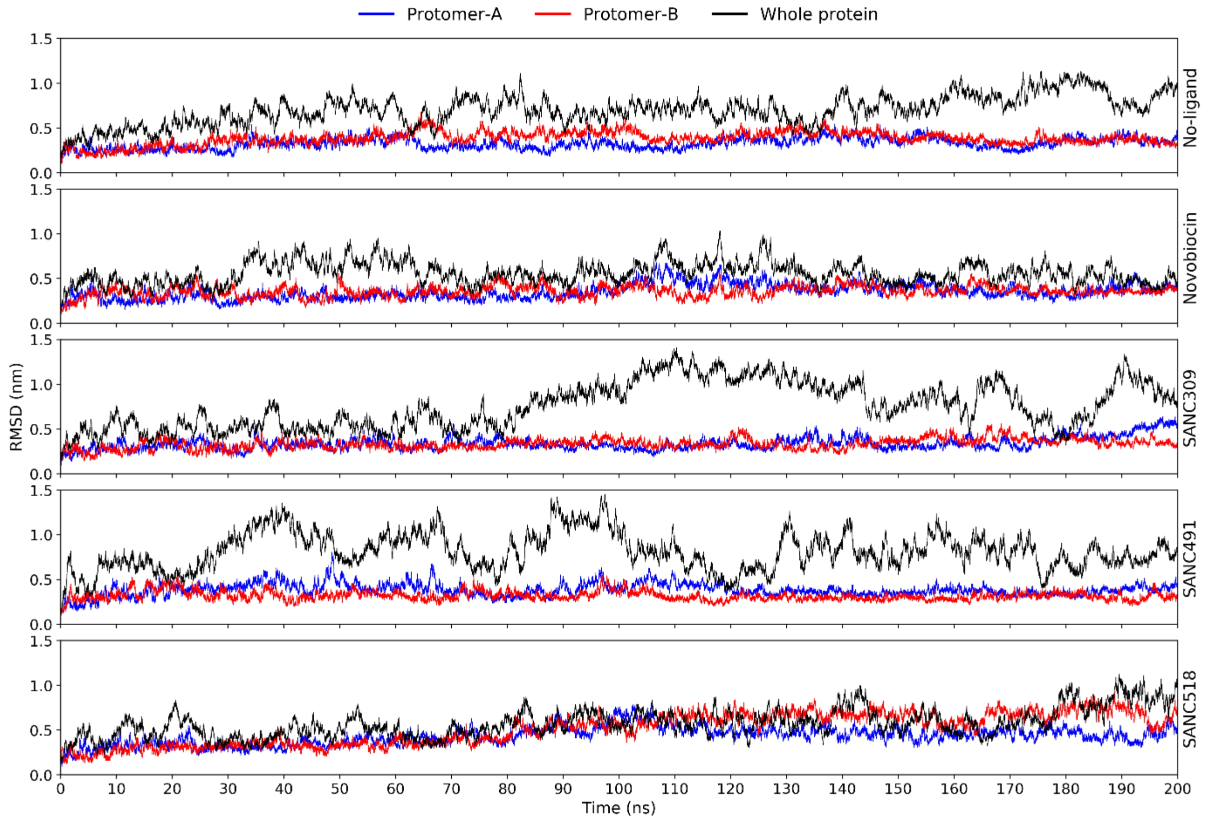

Figure S2: RMSD plots for each ligand bound complex: Black denotes the whole protein backbone RMSD, while blue and red denote the backbone RMSD of each individual protomer

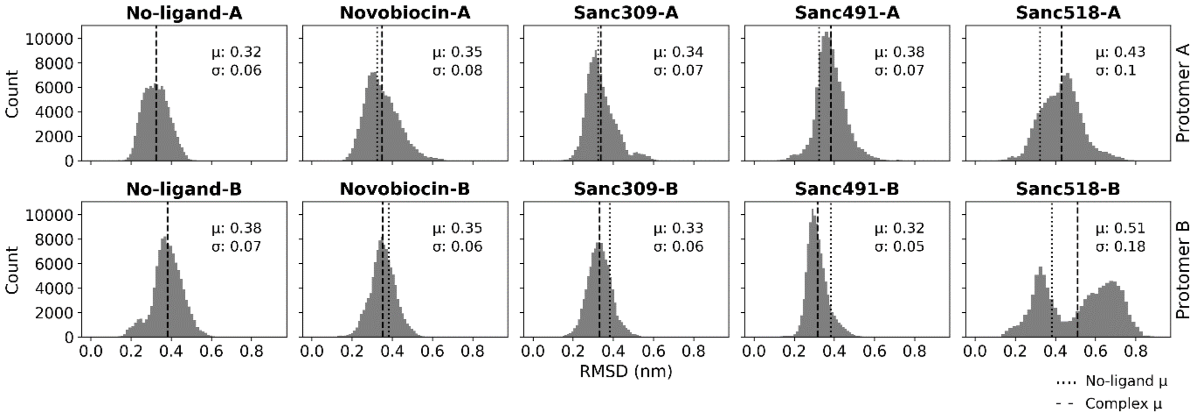

Figure S3: Protomer backbone RMSD distribution plots. Comparison of the histogram distribution plots for each ligand bound complex with the ligand-free state. In each case, the shift in the conformational distribution over the 200 ns MD trajectory can be visually assessed by comparing the mean (μ) of each complex (dashed line) with the ligand-free complex (dotted line).

Table S1: Whole protein and individual protomer RMSD distribution statistics. The means of each ligand bound complex was compared to the ligand-free system using the z-test statistic with $\alpha = 0.05$ and a null hypothesis of $H1 - H2 = 0$. The two-sample KS-test statistic was computed to compare the difference in shape between the ligand un/bound systems. The Wilcoxon stat was used to assess the difference between the ligand un/bound distributions.

	Mean	Variance	Std.	Wilcoxon	*p*	Z-stat	*p*	KS-stat	*p*
**No-ligand**	0.70	0.03	0.16	-	-	-	-	-	-
**Novobiocin**	0.56	0.02	0.12	-201.86	0.00	-219.90	0.00	0.16	0.51
**Sanc309**	0.76	0.07	0.26	33.21	0.00	55.48	0.00	0.18	0.36
**Sanc491**	0.81	0.04	0.21	120.36	0.00	134.08	0.00	0.08	1.00
**Sanc518**	0.57	0.03	0.16	-172.15	0.00	-177.47	0.00	0.10	0.95
**No-ligand-A**	0.32	0.00	0.06	-	-	-	-	-	-
**Novobiocin-A**	0.35	0.01	0.08	59.91	0.00	75.75	0.00	0.14	0.68
**Sanc309-A**	0.34	0.01	0.07	28.37	0.00	42.61	0.00	0.12	0.84
**Sanc491-A**	0.38	0.01	0.07	180.25	0.00	193.62	0.00	0.14	0.68
**Sanc518-A**	0.43	0.01	0.10	242.90	0.00	282.52	0.00	0.18	0.36
**No-ligand-B**	0.38	0.01	0.07	-	-	-	-	-	-
**Novobiocin-B**	0.35	0.00	0.06	-96.29	0.00	-91.41	0.00	0.08	1.00
**Sanc309-B**	0.33	0.00	0.06	-165.11	0.00	-165.15	0.00	0.10	0.95
**Sanc491-B**	0.32	0.00	0.05	-218.05	0.00	-227.32	0.00	0.12	0.84
**Sanc518-B**	0.51	0.03	0.18	150.36	0.00	214.71	0.00	0.20	0.24

Table S2: Inter-protomer distance distribution statistics. The means of each ligand bound complex was compared to the ligand-free system using the z-test statistic with α = 0.05 and a null hypothesis of H1 – H2 = 0. The two-sample KS-test statistic was computed to compare the difference in shape between the ligand un/bound systems. The Wilcoxon stat was used to assess the difference between the ligand un/bound distributions.

	Mean	Variance	Std.	Wilcoxon	*p*	Z-stat	*p*	KS-stat	*p*
**No-ligand**	7.00	0.35	0.59	-	-	-	-	-	-
**Novobiocin**	7.40	0.40	0.63	124.58	0.00	36.14	0.00	0.08	1.00
**SANC309**	6.60	0.40	0.63	-136.46	0.00	36.14	0.00	0.18	0.36
**SANC491**	8.09	0.49	0.70	288.06	0.00	36.14	0.00	0.14	0.68
**SANC518**	7.12	0.66	0.81	39.26	0.00	36.14	0.00	0.10	0.95

Table S3: NTD-CTD distance distribution statistics. The means of each ligand bound complex was compared to the ligand-free system using the z-test statistic with α = 0.05 and a null hypothesis of H1 – H2 = 0. The two-sample KS-test statistic was computed to compare the difference in shape between the ligand un/bound systems. The Wilcoxon stat was used to assess the difference between the ligand un/bound distributions.

	Mean	Variance	Std.	Wilcoxon	*p*	Z-stat	*p*	KS-stat	*p*
**No-ligand-A**	8.21	0.06	0.24	-	-	-	-	-	-
**Novobiocin-A**	8.64	0.24	0.49	243.82	0.00	248.97	0.00	0.12	0.84
**SANC309-A**	8.42	0.67	0.82	133.08	0.00	77.49	0.00	0.16	0.51
**SANC491-A**	8.11	0.97	0.99	30.39	0.00	-30.98	0.00	0.26	0.06
**SANC518-A**	7.90	1.49	1.22	6.06	0.00	-78.17	0.00	0.36	0.00
**No-ligand-B**	7.63	0.10	0.32	-	-	-	-	-	-
**Novobiocin-B**	7.75	0.09	0.29	83.65	0.00	85.86	0.00	0.20	0.24
**SANC309-B**	8.01	1.01	1.01	211.95	0.00	112.62	0.00	0.14	0.68
**SANC491-B**	8.06	0.14	0.37	267.11	0.00	277.49	0.00	0.18	0.36
**SANC518-B**	7.91	0.21	0.46	170.17	0.00	156.74	0.00	0.12	0.84

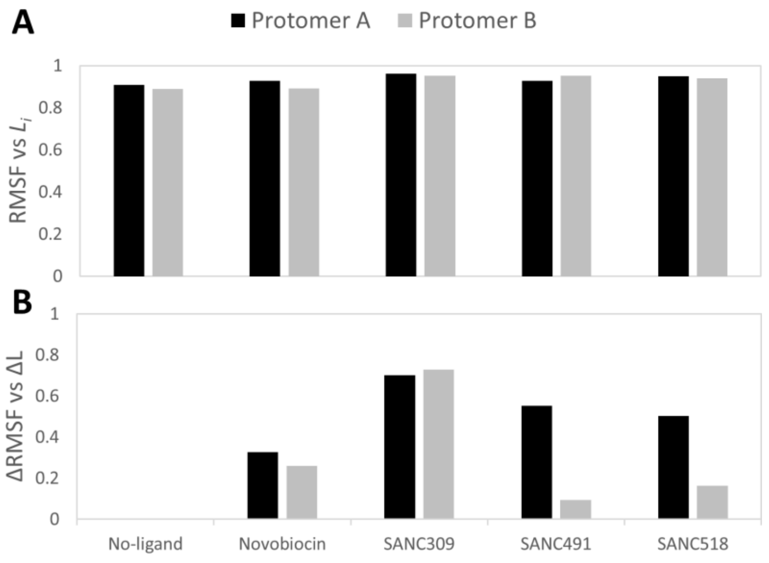

Figure S4: Pearson's correlation coefficients showing the relationship between: (A) L_i vs RMSF; (**B**) ΔRMSF vs ΔL_i

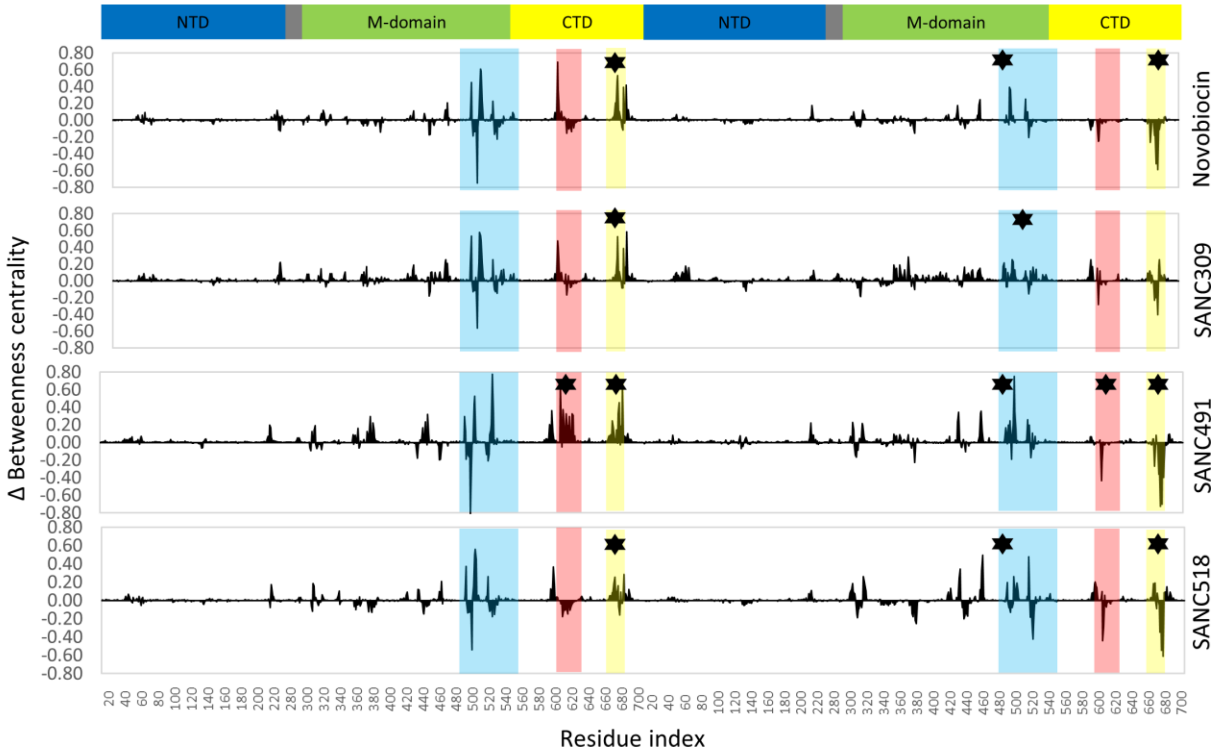

Figure S5: Change in betweenness centrality (ΔBC) for the ligand bound complexes relative to the ligand free system. Shaded regions denote binding site residues: blue sub-pocket, red helix$_2$, yellow four-helix bundle. ★ denote participating ligand interactions at the two ligand binding sites.

A

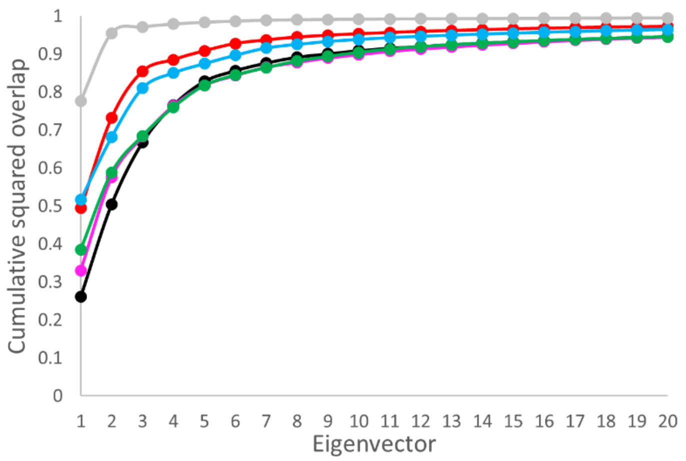

B

Complex	Cosine content for PC1
No-ligand	0.14
Novobiocin	0.17
SANC309	0.31
SANC491	0.13
SANC518	0.37
Known	0.04

Figure S6: (A) Cumulative squared overlap for the first 20 eigenvectors and (B) cosine content for the first eigenvector. Results for the concatenated known conformational states are labelled “Known” and colored grey.

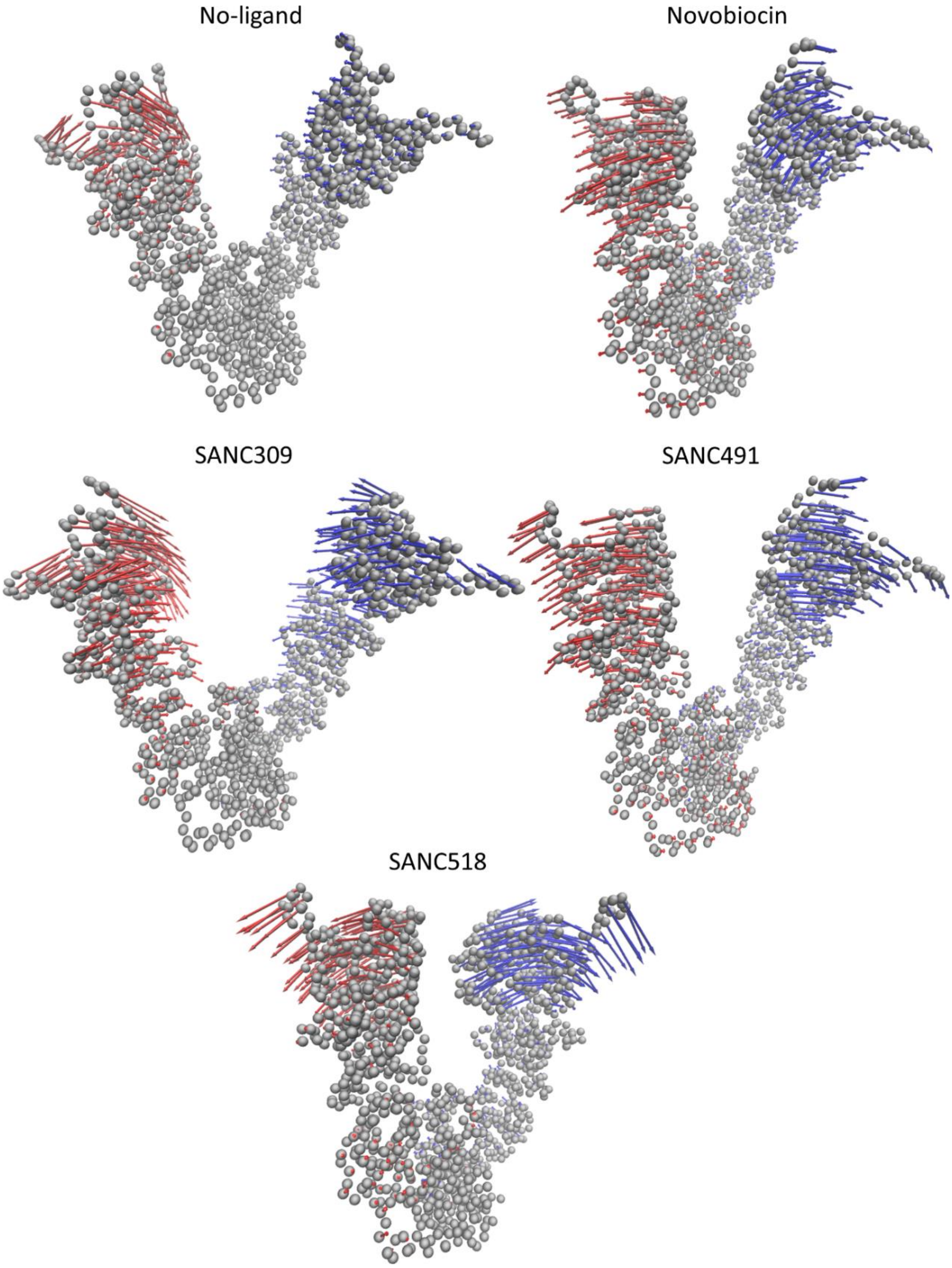

Figure S7: Side view illustrating atomic displacements of first eigenvector of each Hsp90 complex The Hsp90 complex is represented by C_α atoms (grey spheres) and the arrows describe the relative direction and magnitude of the atomic displacements for protomer A (blue) and protomer B (red). Displacement arrows drawn for every 2nd C_α atom to simplify presentation

Movie S1: Correlated motions for the ligand-free complex: depicting dimer closure as protomer A (green) approaches protomer B (cyan)

Movie S2: Correlated motions for the Novobiocin complex: depicting dimer opening as protomer A (green) and protomer B (cyan) displace in opposite directions

Movie S3: Correlated motions for the SANC309 complex: depicting dimer closure as protomer A (green) approaches protomer B (cyan)

Movie S4: Correlated motions for the SANC491complex: depicting dimer opening as protomer A (green) and protomer B (cyan) displace in opposite direction

Movie S5: Correlated motions for the SANC518 complex: depicting dimer opening as protomer A (green) and protomer B (cyan) displace in opposite directions
